# A Benzil- and BODIPY-Based Turn-On Fluorescent Probe for Detection of Hydrogen Peroxide

**DOI:** 10.3390/molecules29010229

**Published:** 2023-12-31

**Authors:** Yunxia Wang, Ye Liu, Bo Liu, Yihua Yuan, Lixia Wei, Mingxiu Wang, Zhe Chen

**Affiliations:** 1 Department of Laboratory Science, Shanxi Medical University, Taiyuan 030001, China; 2 The Sixth Hospital of Shanxi Medical University (General Hospital of Tisco), Taiyuan 030001, China; 3 School of Forensic Medicine, Shanxi Medical University, Jinzhong 030600, China; 4 Key Laboratory of Forensic Toxicology of Ministry of Public Security, Jinzhong 030600, China; 5 School of Public Health, Shanxi Medical University, Taiyuan 030001, China

**Keywords:** turn-on fluorescence, benzil, BODIPY, hydrogen peroxide (H_2_O_2_), vapor detection

## Abstract

Faced with rising threats of terrorism, environmental and health risks, achieving sensitive and selective detection of peroxide-based explosives (PEs) has become a global focus. In this study, a turn-on fluorescent probe (BOD) based on benzil (H_2_O_2_-recognition element) and 4,4-difluoro-4-bora-3*a*,4*a*-diaza-*s*-indacene (BODIPY) derivative (fluorophore) was developed to sensitively and specifically detect hydrogen peroxide (H_2_O_2_). The synthesized BOD had a very weak fluorescence due to intramolecular donor-excited photo-induced electron transfer (*d*-PET) effect; however, it could emit a strong fluorescence since H_2_O_2_ selectively oxidized the benzil moiety and released free BODIPY fluorophore (BOD-COOH). As a result, the proposed BOD detected H_2_O_2_ in linear detection ranged from 25 to 125 µM with a detection limit of 4.41 µM. Meanwhile, the proposed BOD showed good selectivity toward H_2_O_2_, which is not affected by other common reactive oxygen species (ROS) and ions from explosive residues. In addition, a blue shift from 508 to 498 nm was observed in the absorption spectra upon addition of H_2_O_2_. More importantly, the BOD was successfully applied for rapid detection of H_2_O_2_ vapor with good sensitivity (down to 7 ppb), which holds great potential for practical use in public safety, forensic analysis and environmental monitoring.

## 1. Introduction

In recent years, development of simple, sensitive and selective methods for the detection of explosives has become an increasingly critical public concern with rising threats in terrorism, homeland security and environmental pollution. In particular, among the conventional explosives, PEs have attracted widespread attention due to their easy synthesis and high explosive power [1]. However, compared to nitroaromatic explosives, it is difficult to detect PEs because of their own characteristics of lack of nitro groups as well as minimal UV-vis absorption and non-fluorescence [2]. It is well known that H_2_O_2_ has a dual role as a starting synthetic material and a decomposition product of PEs, making it a signature compound for detecting PEs [3]. Thus, achieving highly selective and sensitive detection of H_2_O_2_ is of great significance in effective monitoring and early warning of PEs.

Currently, a variety of analytical methods have been developed for the sensitive detection of H_2_O_2_, including mass spectrometry [4], electrochemical detection [5], colorimetric and fluorimetric assay [6,7,8]. Among them, fluorescence-based probes are the most preferred methods due to their low cost, rapid response, high sensitivity and selectivity [9,10]. Usually, boronate ester chromophore-based fluorescent probes are used based on the cleavage of the boronate group by H_2_O_2_, leading to an increase in fluorescence intensity [11,12,13]. However, these probes were reported to be susceptible to nitric oxide (NO), meaning the detection result can be disturbed by NO that decomposed by the nitro-aliphatic explosives [14]. Therefore, in order to improve the selectivity for PE detection, it is crucial to ensure that the designed probes should be secured from disturbances like nitro-aliphatic and nitro-aromatic explosives as well as their decomposed products. Since Nagano et al. developed a novel fluorescence probe based on benzil chemistry and PET strategy to detect H_2_O_2_ in 2011 [15], many benzil chemistry-based probes have been designed for detection of H_2_O_2_ with a good reaction rate and high specificity [16,17].

As of recently, fluorescent probes based on BODIPY fluorophores hold great promise in explosive detection due to BODIPY’s outstanding photophysical properties, such as high quantum yield and good photo stability [18]. By conjugating the electron-donating group (EDG) or the electron-withdrawing group (EWG) to BODIPY, its photophysical properties can be fine-tuned through PET effect and charge-transfer intersystem crossing (ISC) process, resulting in an increase or suppression of fluorescence emission, which can be used for the construction of turn-on or turn-off fluorescent probes [15,16,17,18,19,20]. Consequently, the combination of benzil chemistry and BODIPY fluorophores can be an appropriate candidate strategy for development of fluorescent probes to detect H_2_O_2_. For example, Li et al. developed a turn-on fluorescent probe (*m*-NBBD) for the visual detection of H_2_O_2_ vapor with high sensitivity and specificity based on BODIPY combined with benzil moiety [21]. Recently, in our previous study, a cancer cell-targeting fluorescence turn-on probe based on a benzil group and a BODIPY derivative was designed for detecting H_2_O_2_ in biological systems, showing a high selectivity that can distinguish H_2_O_2_ from common ROS [22]. As a consequence, and inspired by the excellent selective response of benzil toward H_2_O_2_ and great fluorescence features of BODIPY, in this study, we designed a turn-on fluorescent probe (BOD) for the detection of H_2_O_2_ and H_2_O_2_ vapor by combining the merits of benzil and BODIPY.

It was hypothesized that the proposed BOD could show almost no fluorescence owing to fluorescence quenching via the *d*-PeT process. Upon H_2_O_2_ recognition, benzil could transform to benzoic acid, followed by the release of BODIPY dye, resulting in a fluorescence increment. Consequently, H_2_O_2_ could be evaluated by measuring the BODIPY fluorescence, where benzil acted as the recognition group and BODIPY acted as the fluorophore. Meanwhile, the detection solution color changed from red to yellow-green, displaying potential for colorimetric assay. More importantly, BOD was further applied for the detection of H_2_O_2_ vapor, which brought significant implications for the practice of PE detection.

## 2. Results and Discussion

### 2.1. Principle of the Benzil- and BODIPY-Based H_2_O_2_ Assay

**Scheme 1:**
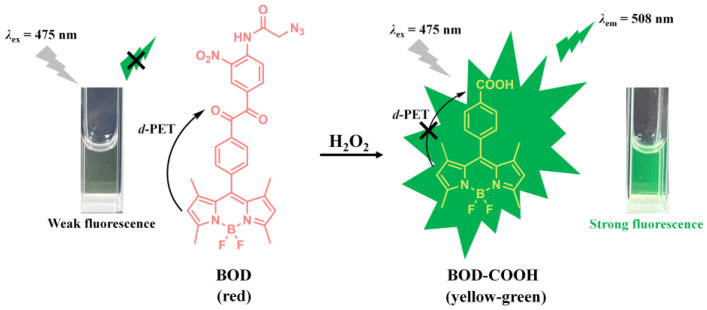
Schematic representation of the benzil- and BODIPY-based turn-on fluorescent probe for the detection of H_2_O_2_ (the X cross indicated that the process did not occur in the corresponding condition).

In this work, a benzil- and BODIPY-based turn-on fluorescent probe that employed benzil as H_2_O_2_-recognition group and BODIPY as fluorophore for the detection of H_2_O_2_ as well as the recognition mechanism of the proposed fluorescent assays is developed and depicted in Scheme 1. Initially, the designed BOD was almost non-emissive, which was attributed to the occurrence of the d-PET effect from BODIPY fluorophore to nitrobenzene [15,22]. In contrast, in the presence of H_2_O_2_, the benzil moiety could be specifically recognized and oxidized by H_2_O_2_, and a BODIPY derivative (BOD-COOH) could be formed and subsequently released, resulting in a turn-on fluorescence signal. Similar working principle had also been reported previously by many researchers [17,23]. Additionally, the detection solution underwent a visual color change from red (BOD) to yellow-green (BOD-COOH), which had the potential for colorimetric analysis of H_2_O_2_. Therefore, highly sensitive and specific detection of H_2_O_2_ can be successfully achieved based on the proposed benzil- and BODIPY-based probe through the fluorescence and colorimetric signal change.

### 2.2. Synthesis and Characterizations of the BOD

**Scheme 2:**
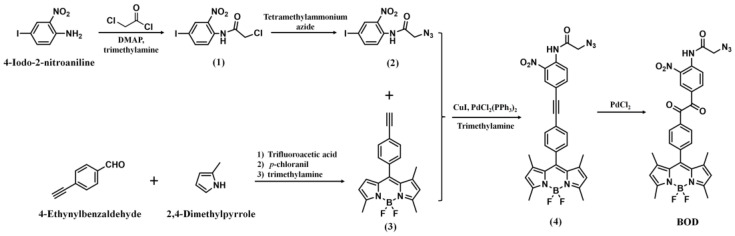
Chemical structure and synthetic route of BOD.

As shown in Scheme 2, BOD was synthesized according to our previous study [22]. First, Compound (**1**) was synthesized through the reaction of 4-iodo-2-nitroaniline and chloroacetyl chloride in the presence of 4-dimethylaminopyridine (DMAP) and trimethylamine. Then, Compound (**1**) was reacted with tetramethylammonium azide to produce azide-modified Compound (**2**). Ethynyl-modified BODIPY of Compound (**3**) was synthesized based on the reported procedures by using 4-ethynylbenzaldehyde and 2,4-dimethylpyrrole, where the reaction was conducted with stirring under nitrogen protection [24]. Subsequently, Compound (**4**) was synthesized via Sonogashira coupling reaction of (**2**) and (**3**) by utilizing PdCl_2_(PPh_3_)_2_, CuI as catalysts and trimethylamine as a base. Finally, the BOD was obtained through the oxidation of (**4**) in the presence of PdCl_2_, and was characterized by ^1^H NMR, ^13^C NMR spectroscopies and high-resolution mass spectrometry (HR-MS) (Figures S1–S3).

### 2.3. Feasibility of the Benzil- and BODIPY-Based H_2_O_2_ Assay

**Figure 1:**
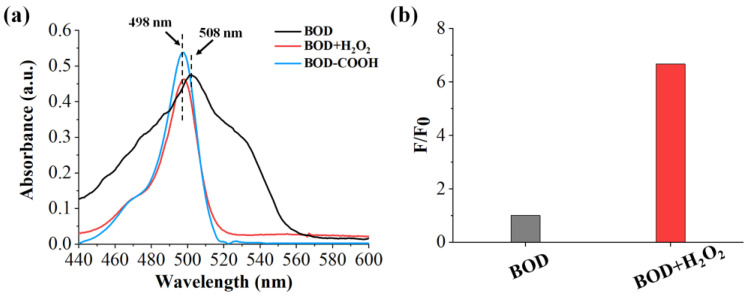
(**a**) UV-Vis absorption spectra of BOD before and after the addition of H_2_O_2_, and the BODIPY derivative (BOD-COOH) in PBS (0.1 M, pH = 7.4, 5% DMSO) buffer ([BOD] = [BOD-COOH] =10 µM, [H_2_O_2_] = 1 mM); (**b**) Fluorescence intensity ratio of the BOD at 508 nm before (F_0_) and after (F) the addition of H_2_O_2_ in PBS (0.1 M, pH = 7.4, 5% DMSO) buffer ([BOD] = 5 µM, [H_2_O_2_] = 300 µM, λ_ex_ = 475 nm, λ_em_ = 508 nm).

**Figure 2:**
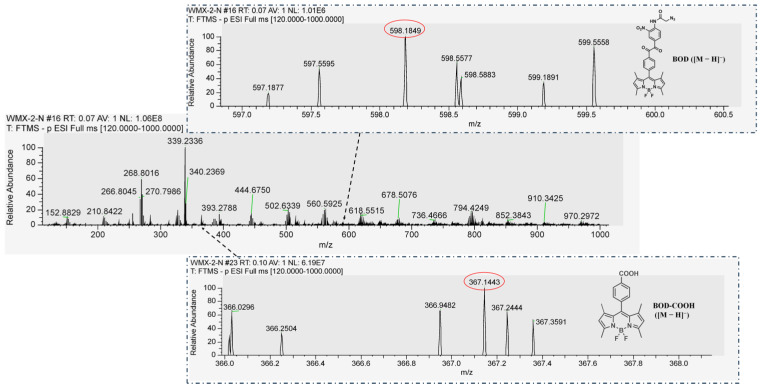
HR-MS analysis showing peaks of the BOD and BOD-COOH after the addition of H_2_O_2_ in a negative ion mode.

The feasibility of the proposed strategy was demonstrated by detecting the absorption spectra and the fluorescence response signals of BOD before and after the addition of H_2_O_2_. As shown in Figure 1a, the BOD probe showed its characteristic absorption profile with an absorption maximum at about 508 nm; however, the addition of H_2_O_2_ to BOD induced a blue shift from 508 nm to 498 nm. More importantly, the absorption spectra of the product of reaction of BOD and H_2_O_2_ was similar to that of the BODIPY derivative (BOD-COOH), confirming that BOD indeed reacted with H_2_O_2_ and generated the product BOD-COOH. Likewise, from Figure 1b, the fluorescence intensity of BOD showed an about seven-fold increment after the reaction of BOD and H_2_O_2_, demonstrating the formation of the BODIPY dye BOD-COOH. In addition, the HR-MS analysis of the probe BOD was conducted before and after reacting with H_2_O_2_. As described in Figure 2, after incubation of the BOD and H_2_O_2_ for 2 h at 37 °C, the HR-MS analysis showed peaks at m/z = 367.1443 and 598.1849, which can be assigned to BOD-COOH ([M − H]^−^) and BOD ([M − H]^−^), respectively, further confirming the reaction of the BOD and H_2_O_2_, and subsequently the generation and release of the BOD-COOH product. Therefore, these results suggested that the proposed BOD probe for the detection of H_2_O_2_ is feasible as it can simultaneously generate fluorescence and colorimetric signal change.

### 2.4. Optimization of the Benzil- and BODIPY-Based H_2_O_2_ Assay Conditions

In order to achieve good performance of the proposed probe BOD for H_2_O_2_ detection, detection solution, incubation time and pH of solution were optimized. It is well known that BODIPYs are sensitive to the solvent [25]; therefore, absorption and fluorescence spectra of BOD were detected in various solvents to study if BOD could form aggregation that could subsequently affect detection efficiency. As shown in Figure S4, the absorption and fluorescence spectra measurements of BOD were determined in DMSO, PBS (0.1 M, pH = 7.4, 5% DMSO) and PBS (0.1 M, pH = 7.4, 1% DMSO) buffer, respectively. The absorption spectrum of BOD in DMSO exhibited characteristic absorption maxima at about 498 nm, while the maximum absorption peaks of BOD in PBS (0.1 M, pH = 7.4, 5% DMSO) and (0.1 M, pH = 7.4, 1% DMSO) buffer were, respectively, at about 508 and 520 nm (Figure S4a). Moskalensky et al. found that BODIPY-based dyes aggregate in water (1% DMSO), which simultaneously showed a wide absorption peak (at about 500 nm) and two fluorescence peaks [26]. As for the two fluorescence peaks, one at about 500 nm corresponded to the emission band of BODIPY in organic solvents, and one at about 650 nm corresponded to the formation of aggregation. However, the BOD only had one fluorescence peak at about 508 nm in both PBS (5% DMSO) and PBS (1% DMSO) buffer solutions (Figure S4b), indicating that the BOD was completely dissolved and no obvious aggregation phenomenon was observed in both PBS buffer solutions. In Figure S4a, the BOD shows wide absorption in the PBS buffer solution, which can be attributed to the influence of the contained benzil and azido group, and similar results have also been observed in the study of benzi by Mittal [27]. In addition, Nagano et al. designed and synthesized a library of BODIPY-based environmental polarity sensors by utilizing photoinduced electron-transfer-controlled fluorescence on/off switching. They demonstrated that the fluorescence property can be well controlled by the PET mechanism, which depends on solvent polarity [28]. Thus, as shown in Figure S4b, the fluorescence intensity of BOD in DMSO was stronger than that in the PBS (5% DMSO and 1% DMSO) buffer, which can be explained by the change in solvent polarity, resulting in a different PET process and fluorescence intensity. Consequently, the fluorescence emission of BOD was quenched mainly by the PET pathway in PBS (5% DMSO and 1% DMSO) buffer, which was not due to aggregation.

**Figure 3:**
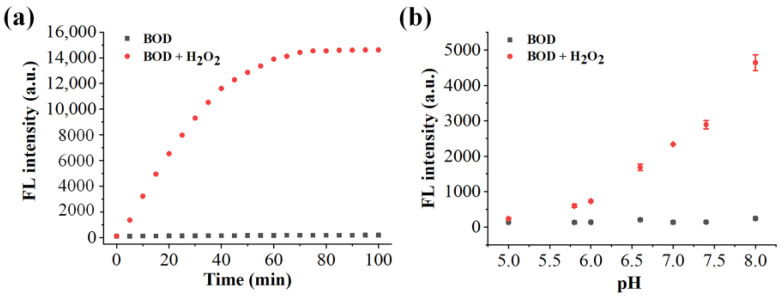
(**a**) Fluorescence intensity change in BOD at 508 nm after reaction with H_2_O_2_ for different incubation times ([BOD] = 5 µM, [H_2_O_2_] = 2 mM); (**b**) Fluorescence intensity change in BOD at 508 nm after reaction with H_2_O_2_ at different pH (error bars indicate the standard deviation of measurements (*n* = 3), [BOD] = 5 µM, [H_2_O_2_] = 1 mM).

As for the optimization of incubation time, in Figure 3a, the BOD fluorescence increased on increasing the incubation time for the reaction of BOD and H_2_O_2_, with the fluorescence reaching a maximum at 60 min, and remained stable. Thus, 60 min was selected as the optimal incubation time.

In addition, the effect of pH was also investigated by detecting the BOD fluorescence in the presence of H_2_O_2_ in PBS buffer from pH 5.0 to 8.0. After reaction with H_2_O_2_, the BOD fluorescence was pH-dependent and rose with increasing pH value as shown in Figure 3b, which can be attributed to the deprotonation of carboxylic acid (product BOD-COOH) to form carboxylate (−COO^−^) in alkaline solution [21]. In contrast, the fluorescence of free BOD remained stable between pH values of 5.0 and 8.0. Considering the generality of the proposed approach, therefore, physiological pH (pH = 7.4) in PBS buffer was chosen for the following experiments.

### 2.5. Photostability Study of the BOD Probe

**Figure 4:**
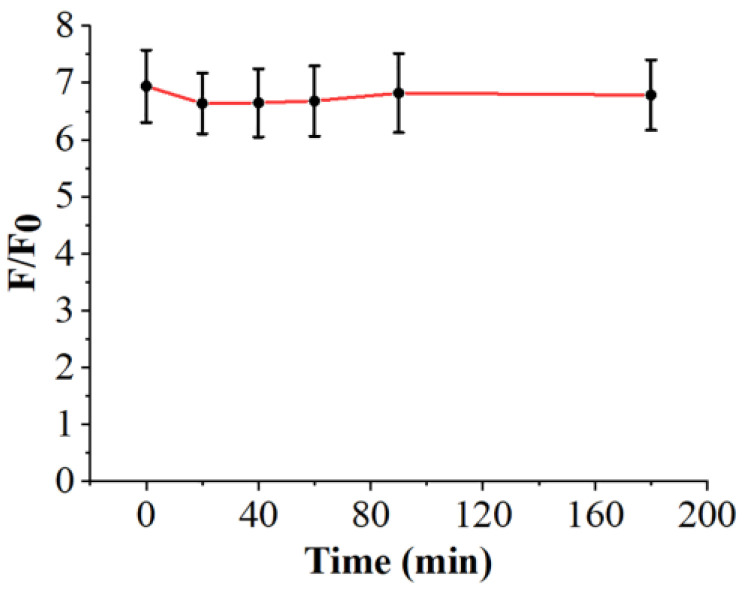
Photostability was evaluated by detecting the fluorescence intensity ratio of BOD at 508 nm before (F_0_) and after (F) the addition of H_2_O_2_ upon continuous UV irradiation (2 × 8 W, emission at 365 nm) for 180 min ([BOD] = 5 µM, [H_2_O_2_] = 300 µM).

Further, the photostability of the proposed BOD probe was studied since it is vital for optical-based sensing probes. In particular, PEs were usually detected by H_2_O_2_ that generated by their photolysis. As a result, photostability is a critical requirement for the design of probes to detect H_2_O_2_. It was found that the fluorescence response of BOD remained stable in the presence of H_2_O_2_ upon continuous UV irradiation at 365 nm for 180 min (Figure 4), indicating great photostability of the proposed BOD.

### 2.6. Sensitivity of the Benzil- and BODIPY-Based H_2_O_2_ Assay

**Figure 5:**
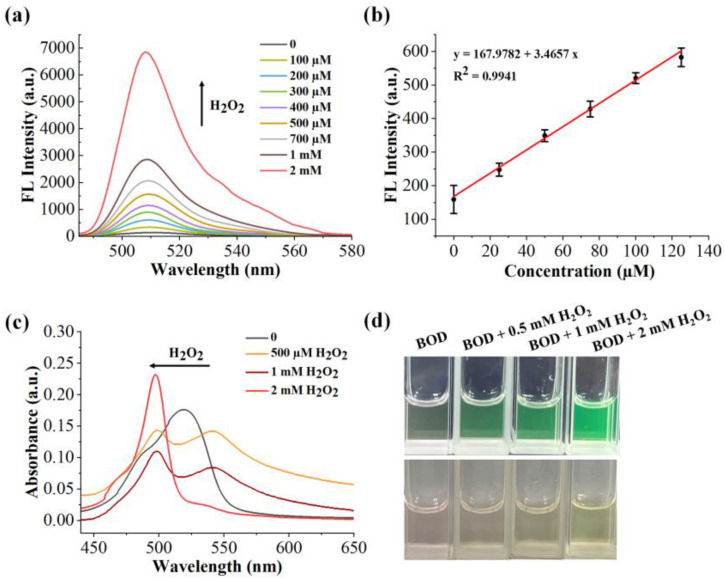
(**a**) Fluorescence response spectra of the proposed BOD probe with different concentrations of H_2_O_2_ in the range of 0–2 mM; (**b**) The relationship between the BOD fluorescence intensity at 508 nm and different concentrations of H_2_O_2_ in the range of 0 to 125 µM (0, 25, 50, 75, 100, 125 µM); (**c**) UV−vis absorption spectra of the BOD with different concentrations of H_2_O_2_; (**d**) Upper panel: the photographs of the BOD before and after addition of H_2_O_2_ under a UV lamp; Bottom panel: the photographs of the BOD before and after addition of H_2_O_2_ under visible-light irradiation. [BOD] = 5 µM.

In order to evaluate the H_2_O_2_ detection capability of the proposed BOD probe, the fluorescence spectra of the BOD were measured in the presence of H_2_O_2_ at different concentrations (0, 0.1, 0.2, 0.3, 0.4, 0.5, 0.7, 1, 2 mM). As the concentration of H_2_O_2_ increased from 0 to 2 mM, the fluorescence emission peak of BOD was gradually enhanced (Figure 5a), which was consistent with the fact that H_2_O_2_ could selectively oxidize the benzil moiety of BOD and generate the BODIPY fluorophore BOD-COOH, subsequently resulting in a turn-on fluorescence signal with a bright green fluorescence under UV radiation (Figure 5d, upper panel). Furthermore, it was observed that the BOD fluorescence intensity at 508 nm had a good linear correlation to the concentration of H_2_O_2_ in the range of 0 to 125 µM with a correlation coefficient of 0.9941 (Figure 5b), and a limit of detection (LOD) of 4.41 µM was determined according to 3σ/K (σ is the standard deviation and K is the slope of the curve of linear regression).

In addition, the colorimetric sensing ability of the proposed probe BOD was evaluated by measuring UV-vis absorption spectra of the BOD with different concentrations of H_2_O_2_. The results showed that the characteristic absorption peak of BOD at 508 nm disappeared upon the addition of H_2_O_2_, while two new peaks appeared at ~540 nm and ~498 nm (Figure 5c). And the peak at ~540 nm disappeared gradually with the increase in H_2_O_2_ concentration, but at the same time, the peak of BOD at ~498 nm displayed an enhancement. The generation of the new peak at ~540 nm might derive from the intermediate product of reaction between BOD and H_2_O_2_ [15,21]. Meanwhile, the color of the above BOD solution was clearly changed from red to yellow-green after H_2_O_2_ treatment with a certain concentration (Figure 5d, bottom panel). As a result, H_2_O_2_ can be visually detected by the naked eye when its concentration exceeds 0.5 mM.

### 2.7. Selectivity of the Benzil- and BODIPY-Based H_2_O_2_ Assay

**Figure 6:**
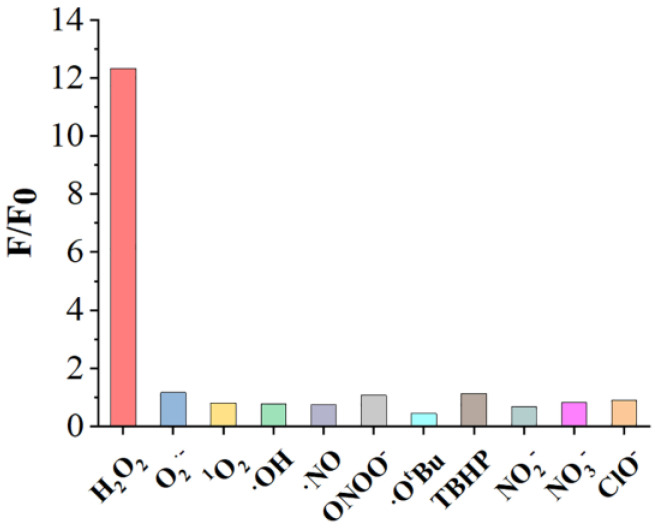
Selectivity assay of fluorescence responses of the BOD to H_2_O_2_ and other ROS (O_2_^•−^, ^1^O_2_, •OH, •NO, ONOO^−^, •O^t^Bu, and TBHP) and common ions from explosive residues (NO_2_^−^, NO_3_^−^ and ClO^−^). F was the fluorescence intensity of the BOD at 508 nm with the addition of different testing species, F_0_ was the fluorescence intensity of the BOD at 508 nm without the addition of the testing species; [BOD] = 5 µM.

Selectivity is another important factor for fluorescent probes with reliable and high detection efficiency; therefore, the specificity of the BOD was then investigated by comparing H_2_O_2_ with other common ROS (superoxide radical (O_2_^•−^), ^1^O_2_, •OH, •NO, ONOO^−^, tert-butoxy radical (•O^t^Bu) and tert-butyl hydroperoxide (TBHP)) and common ions from explosive residues (NO_2_^−^, NO_3_^−^ and ClO^−^). As displayed in Figure 6, only H_2_O_2_ was able to induce the highest fluorescence response when compared with other ROS and interferences from the explosive residues. The results indicated that the proposed BOD is capable of detecting H_2_O_2_ with high specificity.

Additionally, reduction in azide-functionalized fluorophores to amines can be utilized for the detection of hydrogen sulfide (H_2_S) [29,30,31,32]; therefore, the BOD was treated with H_2_S to further study whether there is any possible interference of H_2_S with the detection performance of the proposed strategy. As shown in Figure S5a, the BOD showed a relatively weak fluorescence emission in the presence of H_2_S (Figure S5a), while the introduction of H_2_O_2_ induced a large fluorescent enhancement (about 12-fold) (Figure S5b). The reason for this result may be that the PET effect from BODIPY to benzil moiety still remains after the reduction in the azide group as the BOD in the study was constructed by modifying BODIPY with an azide-substituted benzil group, which cannot recover the BODIPY fluorescence, further confirming the selectivity of the proposed BOD for H_2_O_2_ detection.

### 2.8. Detection of H_2_O_2_ Vapor Based on the Proposed BOD Probe

**Figure 7:**
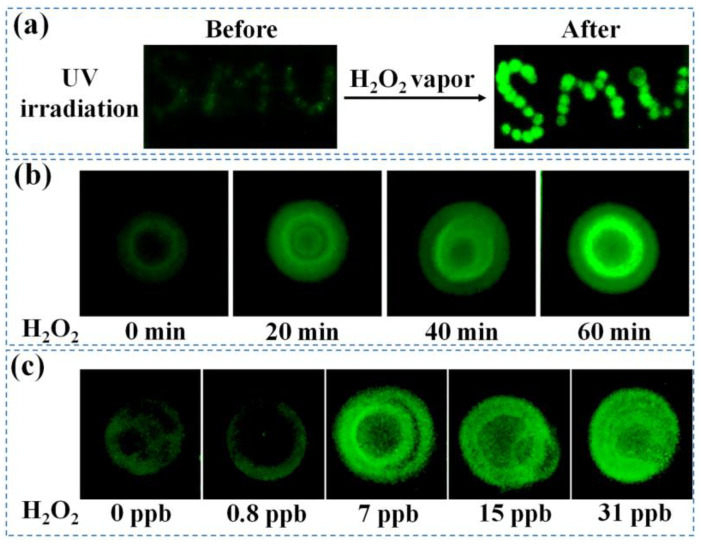
(**a**) The emissive response of the BOD-coated TLC plate with the image of SMU (an abbreviation of Shanxi Medical University) before and after being exposed to H_2_O_2_ vapor under UV irradiation (365 nm); (**b**) The emissive response of the BOD-coated TLC plate toward H_2_O_2_ vapor with different exposure time under UV irradiation (365 nm); (**c**) The emissive response of the BOD-coated TLC plate toward H_2_O_2_ with different concentrations of H_2_O_2_ vapor under UV irradiation (365 nm).

It is well known that trace detection of vapor emanated from explosives is a significant practical application for explosive monitoring. Therefore, the practical applicability of the proposed BOD probe was examined by detecting H_2_O_2_ vapor. We found that the BOD-coated thin-layer chromatography (TLC) plate with the image of SMU (an abbreviation of Shanxi Medical University) emitted an obvious green fluorescence under H_2_O_2_ vapor (Figure 7a), suggesting the success of detection of H_2_O_2_ vapor based on the BOD. Then, the response time of BOD toward H_2_O_2_ vapor was tested, as shown in Figure 7b; a bright green emission could be observed 20 min after exposure to H_2_O_2_ vapor and the green fluorescence increased gradually with exposure time, which can be clearly distinguished from background emission (without H_2_O_2_ treatment), indicating a great potential in H_2_O_2_ vapor rapid detection.

What is more, we studied the sensitivity of the BOD probe for the detection of H_2_O_2_ vapor by being exposed to different concentrations of H_2_O_2_ vapor from 0.8 to 31 ppb (Figure 7c). It was observed that the fluorescence emission of BOD became stronger progressively with increasing the concentration of H_2_O_2_, and the fluorometric change can be easily observed by the naked eye when H_2_O_2_ vapor concentration was above 7 ppb, suggesting the detection range of 0.8 to 31 ppb with the limit of detection of 7 ppb. All these results demonstrated the high sensitivity of the proposed probe for the detection of H_2_O_2_ vapor, implying the proposed assay has great potential for practical applications, especially in rapid on-site detection of PEs.

Although the proposed method showed enough sensitivity, there is still great potential to improve detection time. Nagano et al. developed a series of fluorescence probes based on benzil chemistry and photo-induced electron transfer (PET) strategy to detect H_2_O_2_. They found that the reactivity between benzil and H_2_O_2_ could be adjusted by modification of the benzene ring of benzil [15]. They synthesized five derivatives with various electron-donating or -withdrawing substituents on the benzil moiety, and the results showed that compounds with strongly electron-withdrawing groups of the cyano group and the nitro group exhibited a rapid fluorescence increase even at low concentrations of H_2_O_2_. In addition, they designed the probe by using the water-soluble fluorophore carboxyfluorescein. Thus, the cyano group, fluorescein and rhodamines can be utilized to construct benzil-based fluorescent probes to further optimize the measurement time for the detection of H_2_O_2_.

## 3. Materials and Methods

### 3.1. Materials

All chemicals and solvents were used as received without further purification unless specified otherwise. Chloroacetyl chloride, 4-iodo-2-nitroaniline, trifluoroacetic acid and dimethyl sulfoxide (DMSO) were purchased from Aladdin Chemical Reagent Co., Ltd. (Shanghai, China). Trimethylamine, DMAP, PdCl_2_(PPh_3_)_2_, CuI and PdCl_2_ were purchased from Sigma-Aldrich (St. Louis, MO, USA). 4-ethynylbenzaldehyde and 2,4-dimethylpyrrole were obtained from TCI (Shanghai) Development Co., Ltd. (Shanghai, China). Tetramethylammonium azide, Compounds (**1**), (**2**), (**3**) and (**4**) were synthesized according to previous procedures [20,22]. Standard solutions of NO_2_^−^, NO_3_^−^ and ClO^−^ were purchased from Beijing Mreda Technology Co., Ltd. (Beijing, China). Silica gel TLC plates were purchased from Qingdao Ocean Chemicals (Qingdao, China). All the solvents of analytical grade were employed without further purification.

### 3.2. Apparatus

^1^H and ^13^C NMR spectra were recorded on a JEOL JNM-LA400. High-resolution mass spectra (HR-MS) were conducted on Q Exactive Focus MS (ThermoFisher, Waltham, MA, USA) and MALDI-TOF-MS (Bruker Autoflex III, Billerica, MA, USA).

### 3.3. Synthesis of BOD

PdCl_2_ (0.023 g, 0.132 mmol) and Compound (**4**) (0.05g, 0.088 mmol) were added to 5 mL anhydrous DMSO solution, then the mixture was stirred at 65 °C for 3.5 h under a N_2_ atmosphere. After cooling to room temperature, CH_2_Cl_2_ was added to the mixture. The mixture was subsequently washed with water and dried over MgSO_4_. After the evaporation of solvent, the crude product was purified by a silica gel column using petroleum ether/ethyl acetate (*v*/*v* 4:1) as eluent to produce BOD as a reddish brown solid (0.027 g, 52.1% yield). ^1^H NMR (400 MHz, CDCl_3_): δ (ppm) 11.46 (s, 1H), 9.03 (d, *J* = 9.0 Hz, 1H), 8.93 (d, *J* = 1.9 Hz, 1H), 8.33 (dd, *J* = 8.9, 2.0 Hz, 1H), 8.15 (d, *J* = 8.2 Hz, 2H), 7.52 (d, *J* = 8.2 Hz, 2H), 6.00 (s, 2H), 4.28 (s, 2H), 2.56 (s, 6H), 1.38 (s, 6H). ^13^C NMR (101 MHz, CDCl_3_): δ (ppm) 191.34, 189.50, 166.29, 156.36, 142.68, 142.36, 139.12, 138.51, 136.53, 136.09, 132.68, 130.79, 130.60, 129.24, 128.31, 128.00, 122.16, 121.67, 53.36, 14.72. HR-MS (ESI): *m*/*z*, 598.1849 [M − H]^−^, calcd for C_29_H_24_BF_2_N_7_O_5_, 599.19.

### 3.4. Feasibility of the BOD-Based H_2_O_2_ Assay

BOD was dissolved in DMSO to make a 5 mM stock solution, and standard solution of H_2_O_2_ (30 wt%) was diluted with a PBS buffer to the required concentration. The experiments were performed in 200 µL of PBS buffer containing BOD and different concentrations of H_2_O_2_. After incubation of 60 min, the BOD absorption and fluorescence spectra were recorded using a Tecan plate reader (Tecan, Männedorf, Switzerland) with a Costar 96 Flat Bottom White plate and a Costar black bottom 96-well plate, respectively. All absorption and fluorescence spectra measurements were performed in PBS (0.1 M, pH = 7.4, 5% DMSO) buffer at 37 °C.

### 3.5. Sensitivity and Selectivity of the BOD-Based H_2_O_2_ Assay

BOD and H_2_O_2_ were mixed in the PBS buffer, and the final concentration of BOD was 5 μM. After being incubated with different concentrations of H_2_O_2_ for 60 min, the BOD fluorescence emission spectra of the mixture were measured with a Tianmei FL970 fluorescence spectrophotometer (Tianmei, Shanghai, China) (475 nm for excitation and 485–580 nm for emission). For UV-Vis absorbance spectra, the mixture was measured using a AOEA560 UV-vis spectrometer (AO YI, Shanghai, China) with a 1 cm optical path length cell.

As for the selectivity of the fluorescence assay, O_2_^•−^, ^1^O_2_, •OH, •NO, ONOO^−^, •O^t^Bu, TBHP, NO_2_^−^, NO_3_^−^ and ClO^−^ were used. Based on the published procedures for the preparation of ROS [22,33,34,35], the ROS stock solutions were first prepared as follows. O_2_^•−^ was prepared from the enzymatic reaction of 23.6 mU/L xanthine oxidase and 1.0 mM hypoxanthine in the presence of catalase (25 μg/mL, which is used as a scavenger of H_2_O_2_). ^1^O_2_ was generated through the reaction of 1.0 mM OCl^−^ (5-fold excess) and 200 μM H_2_O_2_. •OH was produced by the Fenton reaction of 1.0 mM Fe(ClO_4_)_2_ with 200 μM H_2_O_2_ in which Fe(ClO_4_)_2_ was in excess. •O^t^Bu was produced by reaction of 1.0 mM Fe(ClO_4_)_2_ with 200 μM TBHP. •NO was obtained from sodium nitroferricyanide. ONOO^−^ was synthesized by the reaction of NaNO_2_ with H_2_O_2_ in an acidic solution, and its concentration was determined by the absorbance peak at 302 nm. To a solution of BOD (5 μM) in a PBS (0.1 M, pH = 7.4, 5% DMSO) buffer, 500 μM H_2_O_2_, 500 μM of O_2_^•−^, ^1^O_2_, •OH, •NO, ONOO^−^, •O^t^Bu and TBHP, 100 mM of NO_2_^−^, NO_3_^−^ and ClO^−^ were added at 37 °C, respectively. After incubation of 60 min, fluorescence intensities of the BOD were recorded at 508 nm with excitation at 475 nm (all fluorescence spectra were recorded using the Tianmei FL970 fluorescence spectrophotometer with the same detection parameters as described above).

### 3.6. Detection of H_2_O_2_ Vapor

In order to detect the H_2_O_2_ vapor, the BOD solution was drop-coated onto a silica gel TLC plate (2.8 (length) × 2.3 (width) cm) with about 1.0 cm radius circle and subsequently dried for 10 min. The experiment was performed by hanging the prepared BOD-coated TLC plate in the saturated vapor of H_2_O_2_ generated in a 100 mL bottle, where approximately 20 mL of H_2_O_2_ solution (diluted down to various concentrations) was put in and sealed for 12 h to reach the equilibrium vapor pressure. The equilibrium vapor pressure corresponding to a specific diluted concentration of H_2_O_2_ solution was deduced from the reported literatures [21,36,37]. Thus, various diluted concentrations (3.5 wt%, 1.4 wt%, 0.7 wt%, 0.35 wt%) of H_2_O_2_ solution were obtained by diluting the commercial 30 wt% H_2_O_2_ solution with pure water, which produce saturated (equilibrium) vapor pressures of H_2_O_2_ of 10.5 ppm, 4.0 ppm, 1.9 ppm and 1.0 ppm, respectively. Then, the above H_2_O_2_ solutions were diluted with water to obtain the final test vapor with lower concentrations (0.8 ppb, 7 ppb, 15 ppb and 31 ppb). After exposure to the vapor for different time intervals, the fluorescence images of the TLC plates were obtained on a gel imaging system under the excitation at 365 nm. The H_2_O_2_ vapor concentration was calibrated by using the following Equation (1) since a binary system of H_2_O/H_2_O_2_ was employed as a solution:

(1) $$y_{H_{2}O_{2}}=\frac{Y_{H_{2}O_{2}}\times X_{H_{2}O_{2}}\times Ps_{H_{2}O_{2}}}{P},$$

where *y*, *Y*, *X*, *Ps*, and *P* were defined as the molar fraction in the vapor phase, the activity coefficient, the molar fraction in the liquid phase, the vapor pressure of H_2_O_2_ (1.801 mmHg), and atmospheric pressure (1 atm), respectively [38]. Moreover, the activity coefficient of H_2_O_2_ was calculated from the following Equation (2) (Margules’ equation):

(2) $$Y_{H_{2}O_{2}}=e^{\;X_{H_{2}O_{2}}{}^{2}\left(-1.2661\;+\;0.2932\;\times \;X_{H_{2}O}\right)}.$$

After incubation, the fluorescence images of the TLC plates were obtained on gel imaging system (Bio-Rad, Hercules, CA, USA) under the excitation at 365 nm.

## 4. Conclusions

In summary, we developed a turn-on fluorescent probe BOD to sensitively and specifically detect H_2_O_2_. The proposed BOD was designed by integrating benzil moiety with BODIPY derivative, in which benzil was employed as an H_2_O_2_ recognition group and BODIPY was used as fluorophore. The fluorescence and absorbance emission of the BOD could be tuned by adjusting H_2_O_2_ concentration, so H_2_O_2_ can be detected through the fluorescence and colorimetric signal change with high sensitivity and selectivity. More importantly, the probe BOD exhibited fast response (down to 20 min) and high sensitivity (down to 7 ppb) for the detection of H_2_O_2_ vapor, providing great potential for real-time in-field detection and monitoring of PEs.

## Data Availability

Data are available from the authors upon reasonable request.

## Supplementary Materials

The following supporting information can be downloaded at: https://www.mdpi.com/article/10.3390/molecules29010229/s1, Figure S1: 1H NMR spectrum of the probe BOD; Figure S2: 13C NMR spectrum of the probe BOD; Figure S3: HR-MS of the probe BOD; Figure S4: Absorption (a) and fluorescence (b) spectra measurements of BOD were determined in DMSO, PBS (0.1 M, pH = 7.4, 5% DMSO) and PBS (0.1 M, pH = 7.4, 1% DMSO) buffer, respectively. ([BOD] = 10 µM); Figure S5: (a) Fluorescence responses of the proposed BOD to H2O2 and H2S, respectively. (b) Fluorescence intensity ratio (F/F0) changes of the BOD after the addition of H_2_O_2_ and H_2_S, respectively. F was the fluorescence intensity of the BOD at 508 nm with the addition of different targets, F_0_ was the fluorescence intensity of the BOD at 508 nm without the addition of the targets. [BOD] = 5 µM, [H_2_O_2_] = [H_2_S] = 500 µM.
