# Supplementary material for: A Benzil- and BODIPY-Based Turn-On Fluorescent Probe for Detection of Hydrogen Peroxide
Source: Molecules. 2023 Dec 31;29(1):229. doi: 10.3390/molecules29010229 (PMC10780145; doi:10.3390/molecules29010229)
Supplement: Supplementary file 1 [file molecules-29-00229-s001.zip › molecules-2761106-SI.pdf]

Supplementary Materials

A Benzil- and BODIPY-Based Turn-On Fluorescent Probe for Detection of Hydrogen Peroxide

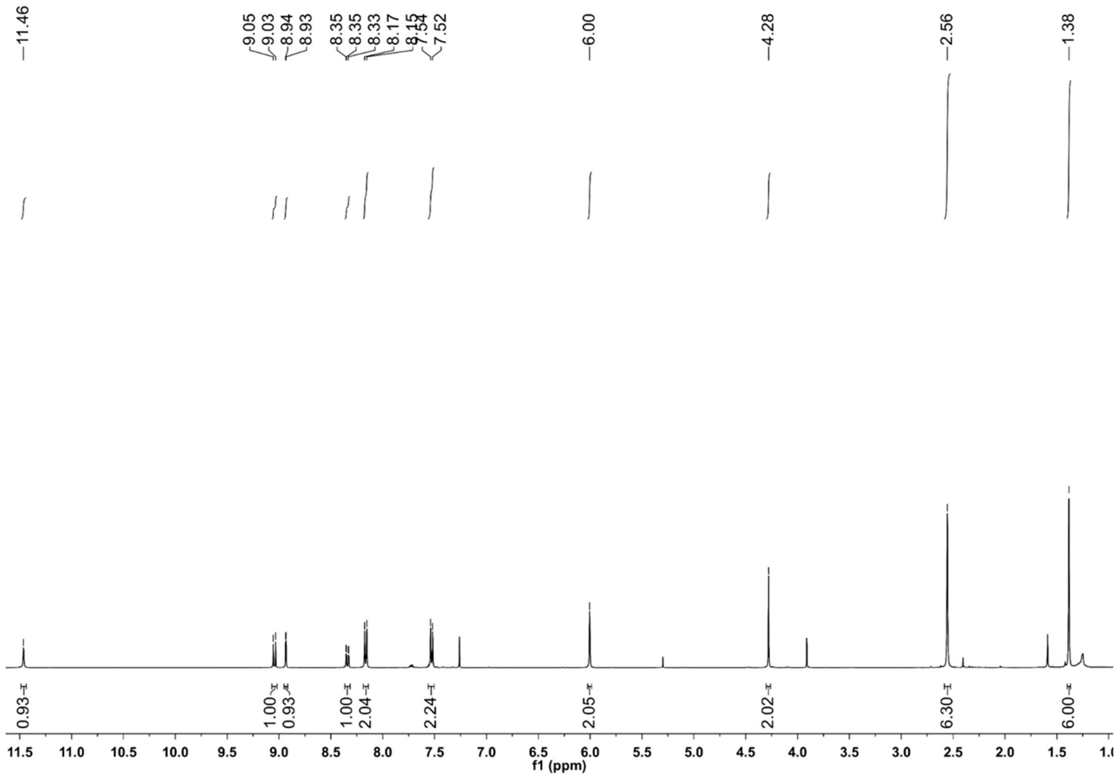

Figure S1. ^{1}H NMR spectrum of the probe BOD.

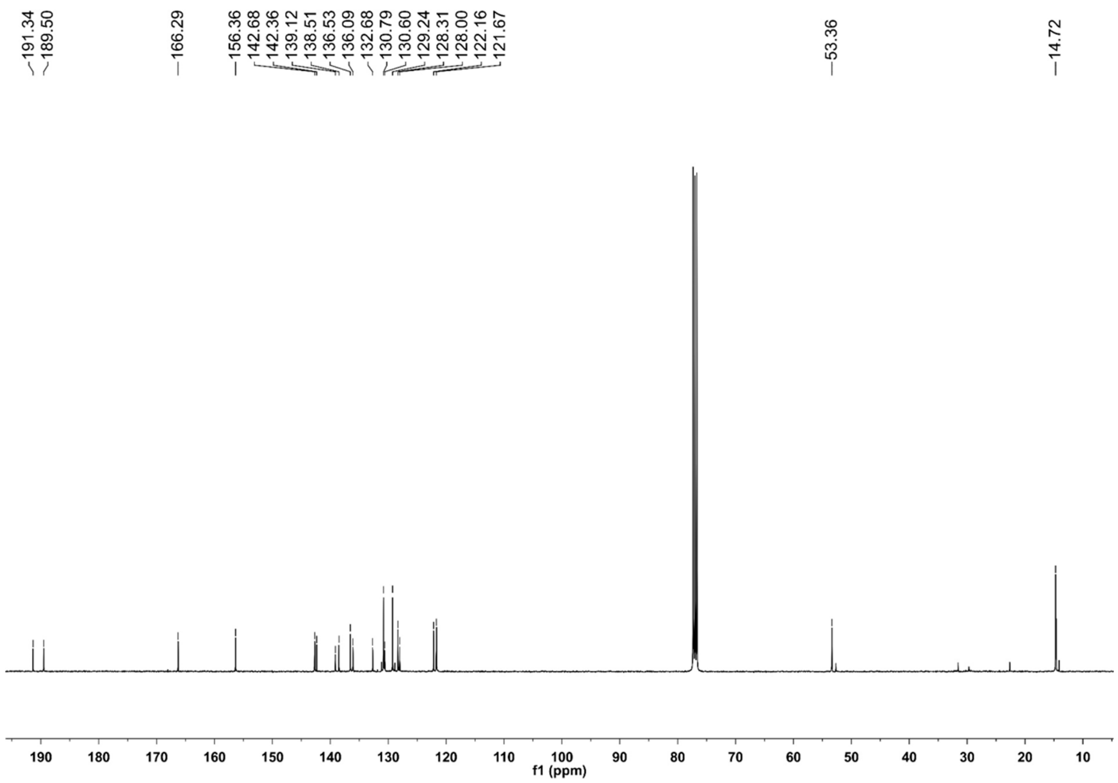

Figure S2. ^{13}C NMR spectrum of the probe BOD.

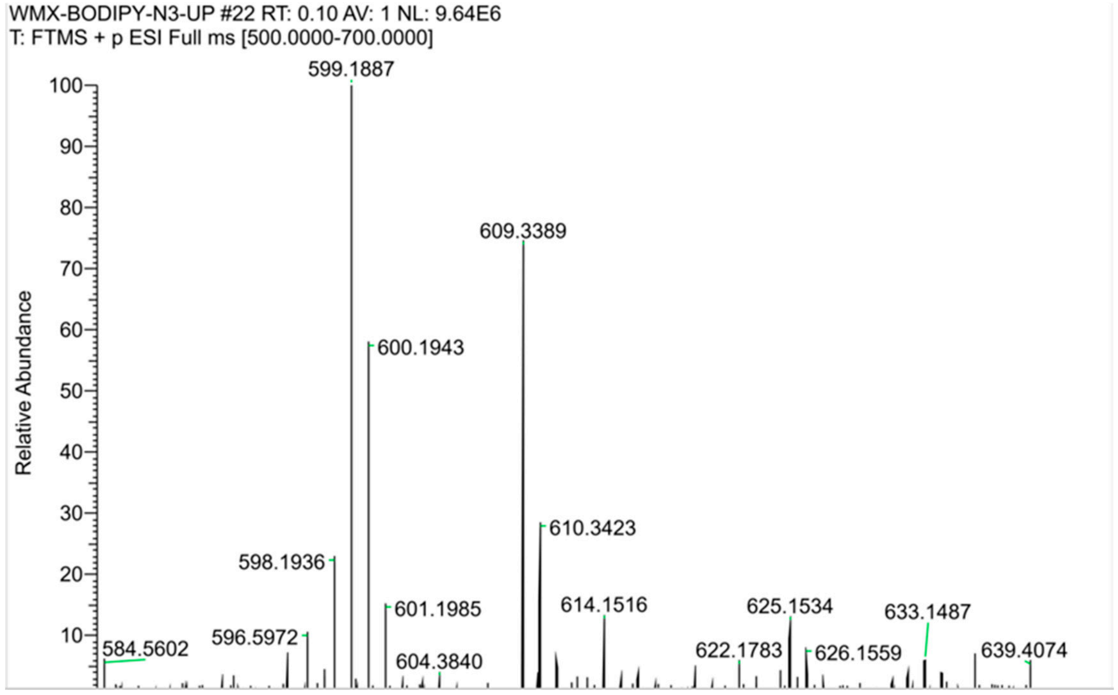

Figure S3. HR-MS of the probe BOD.

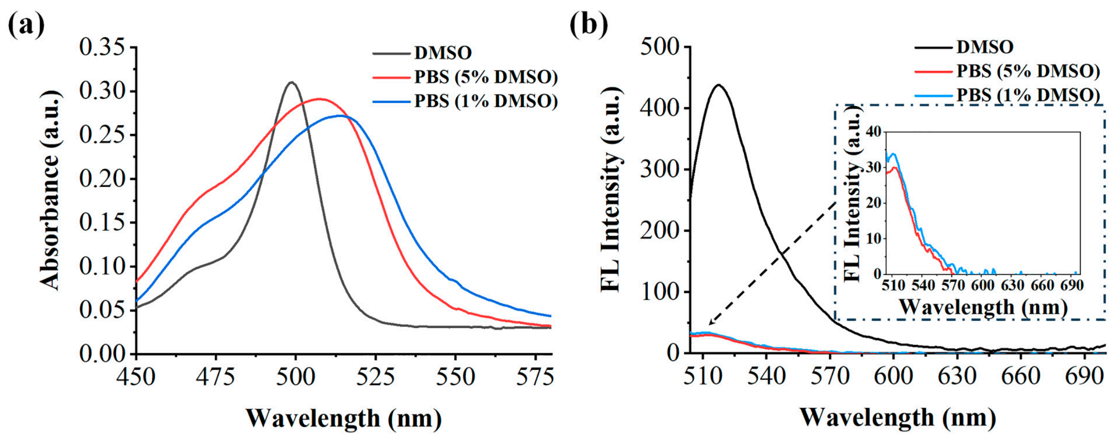

Figure S4. Absorption (a) and fluorescence (b) spectra measurements of BOD were determined in DMSO, PBS (0.1 M, pH = 7.4, 5% DMSO) and PBS (0.1 M, pH = 7.4, 1% DMSO) buffer, respectively. ([BOD] =10 μM)

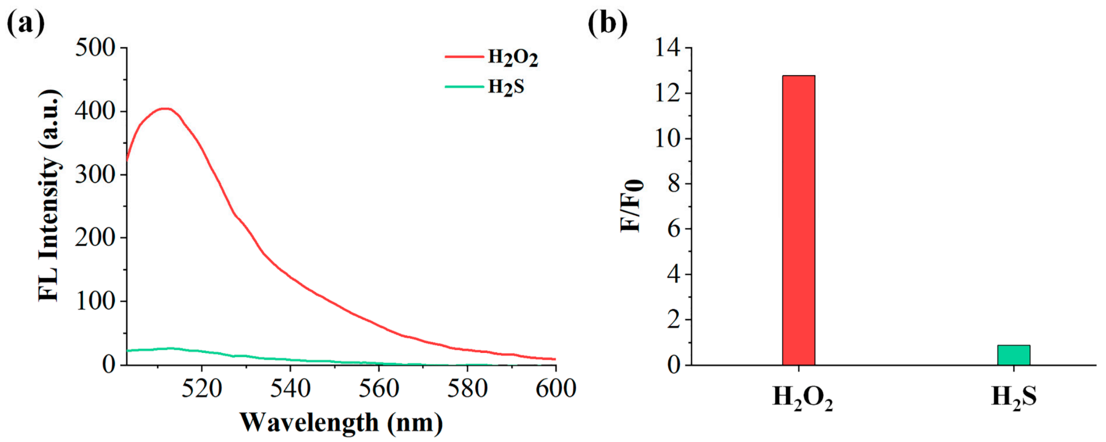

Figure S5. (a) Fluorescence responses of the proposed BOD to H_2O_2 and H_2S, respectively. (b) Fluorescence intensity ratio (F/F_0) changes of the BOD after the addition of H_2O_2 and H_2S, respectively. F was the fluorescence intensity of the BOD at 508 nm with the addition of different targets, F_0 was the fluorescence intensity of the BOD at 508 nm without the addition of the targets. [BOD] =5 μM, [H_2O_2] = [H_2S] =500 μM.
